# The effects of a single night of sleep deprivation on fluency and prefrontal cortex function during divergent thinking

**DOI:** 10.3389/fnhum.2014.00214

**Published:** 2014-04-22

**Authors:** Oshin Vartanian, Fethi Bouak, J. L. Caldwell, Bob Cheung, Gerald Cupchik, Marie-Eve Jobidon, Quan Lam, Ann Nakashima, Michel Paul, Henry Peng, Paul J. Silvia, Ingrid Smith

**Affiliations:** ^1^Defence Research and Development Canada, Toronto Research CentreToronto, ON, Canada; ^2^Department of Psychology, University of Toronto - ScarboroughToronto, ON, Canada; ^3^Naval Medical Research Unit - Dayton, Wright-Patterson Air Force BaseDayton, OH, USA; ^4^Department of Psychology, University of North Carolina at GreensboroGreensboro, NC, USA

**Keywords:** sleep, fatigue, fluency, divergent thinking, executive function

## Abstract

The dorsal and ventral aspects of the prefrontal cortex (PFC) are the two regions most consistently recruited in divergent thinking tasks. Given that frontal tasks have been shown to be vulnerable to sleep loss, we explored the impact of a single night of sleep deprivation on fluency (i.e., number of generated responses) and PFC function during divergent thinking. Participants underwent functional magnetic resonance imaging scanning twice while engaged in the Alternate Uses Task (AUT) – once following a single night of sleep deprivation and once following a night of normal sleep. They also wore wrist activity monitors, which enabled us to quantify daily sleep and model cognitive effectiveness. The intervention was effective, producing greater levels of fatigue and sleepiness. Modeled cognitive effectiveness and fluency were impaired following sleep deprivation, and sleep deprivation was associated with greater activation in the left inferior frontal gyrus (IFG) during AUT. The results suggest that an intervention known to temporarily compromise frontal function can impair fluency, and that this effect is instantiated in the form of an increased hemodynamic response in the left IFG.

## INTRODUCTION

Divergent thinking includes the cluster of “abilities concerned with the ready flow of ideas and with readiness to change direction or to modify information” (Guilford, 1967, p. 139). Researchers have long been interested in how divergent thinking ability is impaired by short-term sleep deprivation, defined as sleep deprivation under 48 h. In a pioneering study on this topic, Horne (1988) found that going 32 h without sleep impaired most aspects of divergent thinking (i.e., fluency, originality, elaboration, and flexibility). Furthermore, this effect was driven not by the participants’ loss of motivation or interest in the tasks, but rather “sleep loss made them fixate on previously successful strategies when attempting solutions to the next problem” (Horne, 1988, p. 535). In other words, sleep deprivation affected cognitive performance in the form of perseveration – defined as “difficulty in changing strategies” (Horne, 1988, p. 530). A subsequent study assessed divergent thinking performance following a single night of sleep deprivation and also demonstrated that it impaired flexibility in divergent thinking – a measure of the conceptual diversity of generated solutions (Wimmer et al., 1992). These early studies converged to demonstrate that short-term sleep deprivation is detrimental for divergent thinking performance.

However, what are the neuroanatomical underpinnings of the impact of short-term sleep deprivation on divergent thinking performance? Much evidence suggests that tasks loading on the prefrontal cortex (PFC) are particularly vulnerable to the impact of sleep loss (Harrison and Horne, 2000; Jones and Harrison, 2001). In this sense, sleep deprivation can be viewed as producing “a reversible functional lesion in the PFC” (Lim and Dinges, 2010, p. 376). Although engagement in divergent thinking activates a distributed network in the brain, functional magnetic resonance imaging (fMRI) studies of divergent thinking have most consistently activated the ventral and dorsal aspects of PFC (Goel and Vartanian, 2005; Fink et al., 2009; Kröger et al., 2012). Activation in these regions has been frequently linked to two processes. First, dorsal and ventral PFC form key regions in the working memory (WM) and executive function systems – necessary for the maintenance and manipulation of information in the focus of attention as well as minimizing distraction during divergent thinking (Gilhooly et al., 2007; Vartanian, 2011; Beaty and Silvia, 2012; De Dreu et al., 2012). This suggests that functional impairment in ventral and/or dorsal PFC due to short-term sleep deprivation could impair divergent thinking by negatively impacting WM and executive function.

Second, consistent with neuropsychological evidence from patient populations (Miller and Tippett, 1996; see also Goel et al., 2013), activation in ventral lateral PFC has been linked to the reduction of constraints that define concepts, thereby facilitating the ways in which they can be manipulated flexibly into new products (Vartanian and Goel, 2005; see also Abraham et al., 2012b). For example, Vartanian and Goel (2005) instructed participants to solve three types of anagrams in the fMRI scanner. On unconstrained trials, they rearranged letters to generate solutions (e.g., Can you make a “Word with ZJAZ”?). On semantically constrained trials, they rearranged letters to generate solutions within particular semantic categories (e.g., Can you make a type of “Music with ZJAZ”?). On baseline trials, they rearranged letters to make specific words (e.g., Can you make the word “JAZZ with ZJAZ”?). The critical comparison of unconstrained vs. semantically constrained trials revealed significant activation in a network including right ventral lateral PFC. Furthermore, a parametric analysis revealed that activation in this region increased as the constraints placed on the anagram search space were reduced across the three trial types. Because optimal performance on divergent thinking tasks necessitates reducing the constraints that define concepts so that they can be manipulated flexibly, functional impairment of ventral lateral PFC because of short-term sleep deprivation could impair divergent thinking by negatively impacting cognitive flexibility.

Consistent with this picture, the results of Gonen-Yaacovi et al.’s (2013) recent large-scale meta-analysis of functional imaging studies of creativity revealed that PFC regions were involved across all task types. The core creativity network was shown to consist of left lateral PFC, associated with various executive processes related to creativity (e.g., fluency, flexibility, inhibition, cognitive control, etc.). Gonen-Yaacovi et al. (2013) noted that these executive processes likely represent components of creative cognition. In addition, the core network included regions involved in the retrieval or formation of remote semantic associations, including the inferior frontal gyrus (IFG), as well as the left angular gyrus and the superior temporal gyrus. These activations were attributed to mechanisms that contribute to both the combination and generation of ideas during creative cognition.

An important recent contribution to this literature was made by Kleibeuker et al. (2013), who examined neural activity in relation to generating alternative uses or ordinary characteristics for common objects. Their results demonstrated that generating alternative uses vs. ordinary characteristics was associated with greater activity in the left angular gyrus, left supramarginal gyrus, and bilateral middle temporal gyrus. However, when they directly compared alternative uses trials in which subjects had generated two or more solutions with trials with zero or one solution, activation was observed in left middle and IFG (pars triangularis). This dissociation suggests that whereas creative idea generation is linked to a primarily left-lateralized parietal and temporal network, the generation of multiple (vs. fewer) creative ideas is associated with activation in left lateral PFC. In other words, engagement in creative cognition *per se* activates a different set of structures than those that are activated when subjects generate multiple ideas. This dissociation is particularly germane to the present study, the focus of which is not creative idea generation but rather *fluency* – defined as the number of generated responses.

Although the aforementioned evidence highlights PFC as key target region where the impact of short-term sleep deprivation on divergent thinking performance might be localized, it is unclear how this effect would manifest itself. In part, this is because the neural effects of sleep loss have been shown to be varied and context-dependent. On the one hand, researchers have observed an elevated hemodynamic response profile [based on the blood-oxygenation level-dependent (BOLD) signal] in relation to verbal learning and logical reasoning following sleep deprivation (Drummond et al., 2000, 2004, 2005; Jonelis et al., 2012). This effect has been interpreted as demonstrating the brain’s compensatory ability to counteract the impairment of normal brain function in the form of increased activity. Furthermore, this compensatory response has been observed most consistently throughout PFC. On the other hand, there is evidence from WM tasks demonstrating that sleep deprivation can in fact lead to a reduction in the BOLD response in PFC (for reviews, see Dang-Vu et al., 2007; Chee and Chuah, 2008). The variability observed in the direction of the effect (i.e., increase or decrease) may be a function of task difficulty. Specifically, the cerebral compensatory response is more likely to be observed in relation to more difficult tasks (Drummond et al., 2004). For the present purposes, we hypothesized that impairment in fluency following short-term sleep deprivation would be accompanied by variation in brain activity in ventral or dorsal PFC, although we did not have an *a priori* prediction regarding the *direction* of the effect.

Furthermore, research on the neuroscience of sleep loss has demonstrated that large individual differences exist in vulnerability to its effects (Caldwell et al., 2005; Chee and Chuah, 2008). For a number of reasons, an individual-differences measure of particular interest to us was fluid intelligence. First, individual differences in fluid intelligence have been shown to predict performance on divergent thinking (and creativity) tasks (Sligh et al., 2005; Nusbaum and Silvia, 2011; Silvia and Beaty, 2012). Second, individual differences in fluid intelligence have been shown to be related to variation in brain activation in ventral and dorsal PFC (Neubauer et al., 2002, 2005; Gray et al., 2003; Lee et al., 2006; Jung and Haier, 2007; Luders et al., 2009; Neubauer and Fink, 2009; Van der Heuvel et al., 2009; Deary et al., 2010). Therefore, we explored the possibility that individual differences in fluid intelligence might influence the impact of sleep deprivation on brain activation in ventral and dorsal PFC during divergent thinking.

Complementing this individual-differences approach, we also explored the possible effects of two self-report measures on brain activation during divergent thinking. The first self-report measure was the Big Five personality factor of openness to experience (John et al., 1991), and the second was the Creative Achievement Questionnaire (CAQ), which measures recognized creative achievements across 10 domains (Carson et al., 2005). They were included because scores on both measures have been shown to be related to performance on divergent thinking tasks (McCrae, 1987; Carson et al., 2005), and could influence the extent to which a participant might be vulnerable to sleep deprivation effects on divergent thinking.

Finally, we were also interested in modeling cognitive effectiveness as a function of daily sleep. To model cognitive effectiveness, each participant in our experiment wore a wrist activity monitor for 7 days (i.e., six nights) prior to each of the fMRI scan sessions. Based on a reduction algorithm, a wrist activity monitor can discriminate a sleeping state from a waking state and thus quantify daily sleep to the nearest minute around the clock, demonstrating significant correlation with polysomnography based on electroencephalography (Dawson and Reid, 1997; Lamond and Dawson, 1999; Arnedt et al., 2001). Actigraphic data were fed into the Fatigue Avoidance Scheduling Tool (FAST^TM^), enabling us to model cognitive effectiveness when scanning for the divergent thinking task was initiated in the scanner. Note that the modeled data do not represent a direct measure of cognitive effectiveness, but rather a derived metric. They are meant to complement our self-report measures of fatigue and sleepiness.

### HYPOTHESES

We conducted an fMRI study to test the following four hypotheses: First, we predicted impairment in fluency following a single night of sleep deprivation compared to a night of normal sleep. Second, we predicted a reduction in modeled cognitive effectiveness following sleep deprivation compared to a night of normal sleep. Third, we predicted that sleep deprivation would impact PFC function in the ventral and/or dorsal regions during divergent thinking, although we had no *a priori* prediction regarding the *direction* of this effect. Fourth, we predicted that the impact of sleep deprivation on PFC function during divergent thinking would be influenced by individual differences in fluid intelligence, openness to experience, and scores on CAQ.

## MATERIALS AND METHODS

### PARTICIPANTS

This study was approved by Defence Research and Development Canada’s Human Research Ethics Committee (DRDC HREC) and Sunnybrook Health Sciences Centre’s Research Ethics Board. The participants were 13 neurologically healthy right-handed volunteers (3 females, 10 males) with normal or corrected-to-normal vision (all determined by medical questionnaire). Average age was 32.23 years (SD = 8.45). The participants received stress remuneration in accordance with DRDC HREC guidelines.

### MATERIALS AND PROCEDURE

After volunteering to participate in this experiment, participants were asked to report to our laboratory 1 week prior to the first fMRI session to be equipped with a wrist activity monitor (www.ambulatory-monitoring.com/motionlogger.html). They were instructed to wear the wrist activity monitor continuously thereafter until arrival at the fMRI facility for the first scan session. Additionally, during this initial session, participants completed paper-and-pencil measures. Our measure of fluid intelligence was *Raven’s Advanced Progressive Matrices* (*RAPM*; Raven et al., 1998). All participants were tested individually. We used a shortened form of the RAPM with 12 problems (Bors and Stokes, 1998). Each participant was given 10 min to complete as many problems as possible (see Vartanian et al., 2013). They also completed the Big Five Inventory (BFI) and the CAQ.

The two fMRI assessments occurred 1 week apart – once following one night of sleep deprivation and once following a night of normal sleep. The order of scans was counterbalanced across participants such that for six participants sleep deprivation occurred prior to the first fMRI scan, whereas for seven participants sleep deprivation occurred prior to the second fMRI scan^1^. Each participant reported to our laboratory for the sleep deprivation session, instructed to arrive at 8 p.m. on the evening prior to the scan. They were instructed not to consume any caffeine, nicotine, or alcohol for 24 h prior to the scan session, were not allowed to leave the laboratory during the sleep deprivation session, and were monitored by staff at all times to ensure that they did not fall asleep^2^. Participants were allowed to read, watch TV, use the telephone, and engage in conversation with research staff. They were provided with two items of food and two beverages during their stay in the laboratory, and could also bring their own snacks and drinks as long as they contained no caffeine, nicotine, or alcohol. They completed the Stanford Sleepiness Scale (SSS) and the Psychomotor Vigilance Task (PVT) hourly between 8 p.m. and 6 a.m. (Hoddes et al., 1973; Dinges and Powell, 1985). On each trial of PVT, participants were instructed to press the spacebar as quickly as possible following the detection of a single target appearing at the center of the computer screen. Participants were brought to the cafeteria for a light breakfast (excluding coffee) prior to departure to the scanning facility (Sunnybrook Health Sciences Centre, Toronto, ON, Canada). In contrast, for scanning following a normal night of sleep, participants were instructed to arrive at our laboratory at 7 a.m. on the day of the scan. They were also instructed not to consume any caffeine, nicotine, or alcohol for 24 h prior to the scan session. All participants were transported by a designated driver, and accompanied by research staff to the scanning facility in order to arrive onsite at 7:30 a.m. All fMRI scans were collected between 8 and 10 a.m.

Upon arrival at the scanning facility, the participants completed the Multidimensional Fatigue Inventory (MFI; Smets et al., 1995) and a brief questionnaire about caffeine, nicotine, and alcohol consumption during the previous 24 h. They were then given instructions about, and examples from, the Alternate Uses Task (AUT). The AUT is a classic and perhaps the most commonly used measure of divergent thinking ability (Guilford, 1967). It instructs participants to generate as many uses as possible for common objects (e.g., brick). In the present study, we only measured fluency, operationalized as the number of generated uses to each prompt. The scanner version of the AUT was modeled after Fink et al. (2009), an identical version of which was administered by our group in a recent fMRI study on divergent thinking (Vartanian et al., 2013). The task was presented in two blocks (i.e., uses and characteristics), the order of which was counterbalanced across participants. Each of the 20 trials in the *uses* block had the same structure. During the *generation* phase, participants were presented with the name of a common object (e.g., knife) and instructed to think of as many uses for it as possible for 12 s. In this phase, the name of the object appeared in black ink. The *response* phase followed immediately afterward during which participants were given 3 s to enter the *number* of generated uses (using an MRI-compatible response pad). In this phase the name of the object appeared in green. Note that in the response phase participants were not instructed to enter the actual uses they had generated, but rather the digit on the keypad corresponding to the number of uses generated in response to the prompt. This color change acted as a prompt to enter the response as quickly as possible^3^. This was followed by an ITI (inter-trial interval) which consisted of three adjacent plus signs (+ + +) varying randomly between 4 and 6 s. Each trial in the *characteristics* block had an identical structure, except that participants were instructed to recall, from long-term memory, physical features characteristic of the object. For example, possible physical features for “knife” could be solid, sharp, metallic, etc.^4^ Note that in the response phase participants were not instructed to enter the actual physical features they had recalled from long-term memory, but rather the digit on the keypad corresponding to the total number of features recalled in response to the prompt.

### fMRI ACQUISITION AND ANALYSIS

A 3-Tesla MR scanner with an eight-channel head coil (Discovery MR750, 22.0 software, GE Healthcare, Waukesha, WI, USA) was used to acquire T1 anatomical volume images (0.86 mm × 0.86 mm × 1.0 mm voxels). For functional imaging, T2^*^-weighted gradient echo spiral-in/out acquisitions were used to produce 26 contiguous 5 mm thick axial slices [repetition time (TR) = 2 s; echo time (TE) = 30 ms; flip angle (FA) = 70°; field of view (FOV) = 200 mm; 64 × 64 matrix; voxel dimensions = 3.1 mm × 3.1 mm × 5.0 mm], positioned to cover the whole brain. The first five volumes were discarded to allow for T1 equilibration effects. The number of volumes acquired was 418 (per session).

Data were analyzed using Statistical Parametric Mapping (SPM8). Head movement was less than 2 mm in all cases. We implemented five preprocessing steps in the following order. We began by slice timing, used to correct for temporal differences between slices within the same volume, using the first slice within each volume as the reference slice. This was followed by realignment and coregistration to ensure that all volumes from the two sessions were realigned to the first volume from the first session. A mean image created from realigned volumes was spatially normalized to the Montreal Neurological Institute echo planar imaging (MNI EPI) brain template using non-linear basis functions. Voxel size after normalization was the SPM8 default, namely 2 mm × 2 mm × 2 mm. The derived spatial transformation was applied to the realigned T2^*^ volumes, and spatially smoothed with an 8 mm full-width at half-maximum (FWHM) isotropic Gaussian kernel. Time series across each voxel were high-pass filtered with a cut-off of 128 s, using cosine functions to remove section-specific low frequency drifts in the BOLD signal. Condition effects at each voxel were estimated according to the general linear model (GLM) and regionally specific effects compared using linear contrasts. The BOLD signal was modeled as a box-car, convolved with a canonical hemodynamic response function. Each contrast produced a statistical parametric map consisting of voxels where the *z*-statistic was significant at *p* < 0.001. Using a random-effects analysis, reported activations survived a voxel-level intensity threshold of *p* < 0.001 (uncorrected for multiple comparisons), and a minimum cluster size of 40 contiguous voxels (Lieberman and Cunningham, 2009; see also Forman et al., 1995).

Using an event-related design, for each session we specified the following regressors corresponding to (1) the generation phase (i.e., uses), (2) the number of uses varying parametrically with the generation phase (first-order polynomial expansion exploring their linear relationship), and (3) the recollection phase (i.e., characteristics), (4) the number of characteristics varying parametrically with the recollection phase (first-order polynomial expansion exploring their linear relationship). Although incorporated into the design, (5) response phase, (6) motor response, and (7) ITI were modeled out of the analyses by assigning null weights to their respective regressors.

## RESULTS

### MANIPULATION CHECKS

We first ascertained the effectiveness of our sleep deprivation procedure by analyzing hourly reaction time (RT) data from PVT (for fatigue) and SSS scores (for sleepiness). For PVT, there was a linear increase in RT throughout the night, *F*(1,11) = 23.07, *p* = 0.001, $\eta_{p}^{2}$ = 0.68. Similarly, for SSS, there was a linear increase in ratings throughout the night, *F*(1,10) = 20.20, *p* = 0.001, $\eta_{p}^{2}$ = 0.67.^5^ Next, we compared MFI data collected immediately prior to entry into the fMRI scanner on both days. The results demonstrated that self-rated fatigue was higher following a night of sleep deprivation than following a night of normal sleep on all subscales of MFI: general [*t*(12) = 4.90, *p* = 0.001, *d* = 1.81], physical [*t*(12) = 2.46, *p* = 0.030, *d* = 0.81], activity [*t*(12) = 3.15, *p* = 0.008, *d* = 0.62], motivation [*t*(12) = 2.58, *p* = 0.024, *d* = 0.73], and mental [*t*(12) = 2.38, *p* = 0.035, *d* = 0.81]^6^. There was no difference in self-reported consumption of caffeine [*t*(8) = -1.98, *p* = 0.083, *d* = -0.79], nicotine [*t*(8) = -1.00, *p* = 0.347], or alcohol [*t*(8) = -1.00, *p* = 0.347] in the 24 h prior to two scan sessions^7^.

### BEHAVIORAL DATA

As predicted, the results demonstrated that participants generated fewer uses for objects (averaged across the uses generated for each of the 20 prompts) following a night of sleep deprivation (*M* = 4.59, SD = 1.70) than following a night of normal sleep (*M* = 5.53, SD = 1.35), *t*(12) = 3.09, *p* = 0.009, *d* = 0.61. Similarly, they recalled fewer characteristics (averaged across the characteristics recalled for each of the 20 prompts) following a night of sleep deprivation (*M* = 4.98, SD = 1.32) than following a night of normal sleep (*M* = 5.86, SD = 1.42), *t*(12) = 2.46, *p* = 0.030, *d* = 0.64. RT was calculated from the beginning of the response phase when the name of the object appeared in green. There was no difference in RT for generating uses for objects after sleep deprivation (*M* = 978 ms, SD = 312) and a night of normal sleep (*M* = 892 ms, SD = 311), *t*(12) = -0.735, *p* = 0.989, *d* = 0.28. Similarly, there were no difference in RT for recalling characteristics following a night of sleep deprivation (*M* = 918 ms, SD = 283) and a night of normal sleep (*M* = 904 ms, SD = 310), *t*(12) = -0.26, *p* = 0.802, *d* = 0.07.

### MODELED COGNITIVE EFFECTIVENESS

**FIGURE 1:**
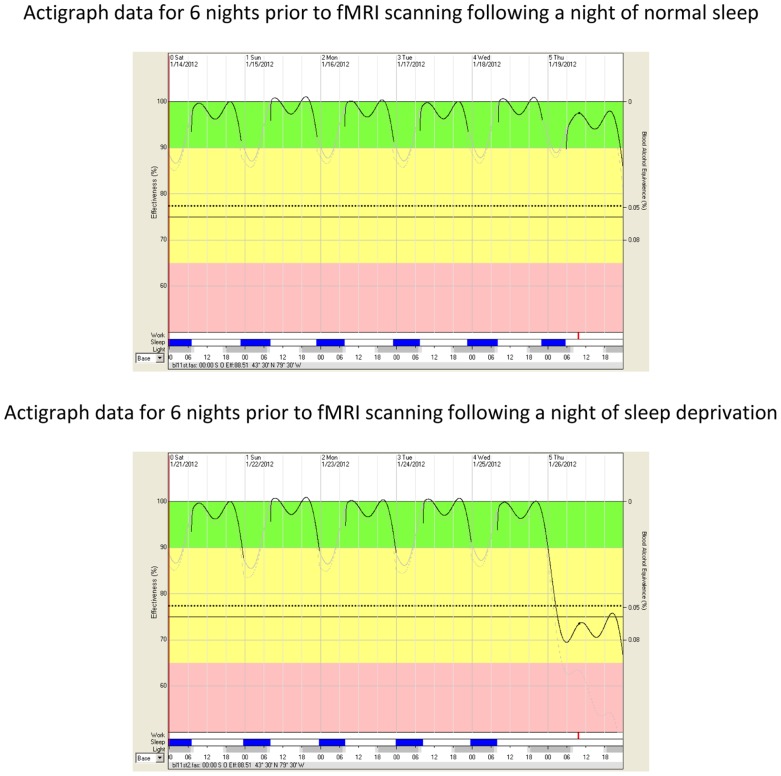
**Impairment in modeled cognitive effectiveness as a function of sleep deprivation.** The vertical axis on the *left* side of the FAST^TM^ graphs represents fitted and transformed modeled cognitive effectiveness as a percentage of optimal performance (100%). The oscillating line in the diagram represents average modeled cognitive effectiveness as determined by time of day, biological rhythms, time spent awake, and amount of sleep. The vertical axis on the *right* side of FAST^TM^ graphs represents the Blood Alcohol Content (BAC) equivalency throughout the spectrum of modeled cognitive effectiveness. A value of 77% modeled cognitive effectiveness corresponds to a blood alcohol content of 0.05% (legally impaired in some jurisdictions). A value of 70% modeled cognitive effectiveness corresponds to a blood alcohol content of 0.08% (legally impaired in most jurisdictions). The dotted line represents the lower 10th percentile of modeled cognitive effectiveness. The green band (from 90 to 100%) represents acceptable modeled cognitive performance effectiveness for workers conducting safety sensitive jobs (e.g., flying, driving, weapons operation, command and control, etc.). The yellow band (from 65 to 90% modeled cognitive effectiveness) indicates caution. Personnel engaged in skilled performance activities such as aviation are recommended not to operate in this bandwidth. The area from the dotted line to the pink area represents the modeled cognitive effectiveness equivalent to the circadian nadir and a second day without sleep. The pink band (below 65%) represents performance effectiveness after two days and one night of sleep deprivation. The abscissa (*x*-axis) illustrates a single 15-min period (red bar) during the fMRI scans for each of the baseline and sleep deprivation conditions as well as sleep timing/duration (blue bars), darkness (gray bars), and time of day in hours. The red bar shows a thickening of the modeled cognitive effectiveness line (immediately above the red bar), reflecting cognitive effectiveness during the fMRI scans.

Modeled cognitive effectiveness (at time of initiating fMRI scanning) was derived by analyzing the FAST^TM^ models which are based on the actigraphically measured sleep data^8^. The FAST^TM^ algorithm is based on variations in time of day, biological rhythms, time spent awake, and amount of sleep. Fitted and transformed FAST^TM^ graphs from a representative participant are illustrated in **Figure 1**. Three participants failed to provide complete actigraph data. Therefore, modeled cognitive effectiveness was computed for the remaining 10 participants. As predicted, modeled cognitive effectiveness was lower at time of fMRI data acquisition following a night of sleep deprivation (*M* = 68.98%, SD = 3.38) than following a night of normal sleep (*M* = 91.36%, SD = 4.72), *t*(9) = 11.58, *p* = 0.000001, *d* = 3.70.

### COVARIATES

Average RAPM score was 8.46 (SD = 1.45). For BFI, the average score for openness to experience was 3.50 (SD = 0.40)^9^. For CAQ, average score was 8.77 (SD = 10.80). There was no correlation between RAPM and openness to experience [*r*(11) = 0.38, *p* = 0.195], RAPM and CAQ [*r*(11) = 0.42, *p* = 0.151], and CAQ and openness to experience [*r*(11) = 0.11, *p* = 0.719]. There were no differences between males and females in RAPM [*t*(11) = 1.09, *p* = 0.299, *d* = 0.87], openness to experience [*t*(11) = 0, *p* = 1, *d* = 0], or CAQ [*t*(11) = 0.10, *p* = 0.923, *d* = 0.08] scores, although our small sample size limits an exploration of sex differences. Although there is evidence to suggest that openness to experience is correlated positively with general intelligence (Ackerman and Heggestad, 1997; Gignac et al., 2004), as well as CAQ (Silvia et al., 2009), the absence of significant correlations among the three variables in the present sample meant that we opted to explore the role of each variable independently on the effect of sleep deprivation on brain function.

### fMRI DATA

**FIGURE 2:**
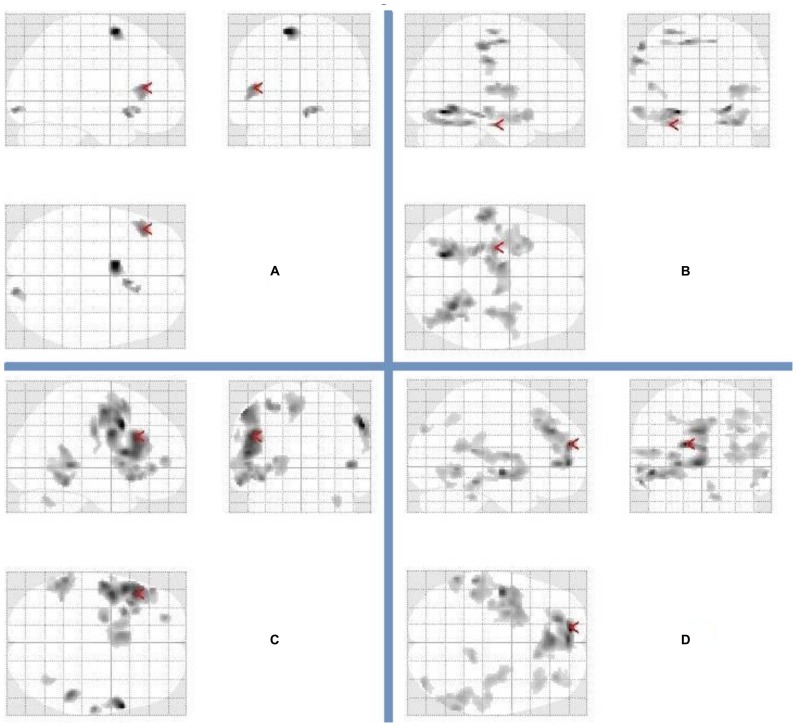
**Patterns of brain activation for divergent thinking (fluency) and recalling characteristics following a night of normal sleep and sleep deprivation.** Glass brains representing activations in relation to **(A)** uses–rest following a night of normal sleep (arrow points to the left inferior frontal gyrus), **(B)** recalling characteristics–rest following a night of normal sleep (arrow points to the left hippocampus), **(C)** uses–rest following sleep deprivation (arrow points to the left inferior frontal gyrus), **(D)** recalling characteristics–rest following sleep deprivation (arrow points to the left medial frontal gyrus). The complete list of activations appears in **Table 1**.

We began our target analyses of interest by first examining the uses–rest (ITI) and recalling characteristics–rest (ITI) contrasts following a night of normal sleep. As can be seen in **Table 1** and **Figure 2**, the uses–rest and recalling characteristics–rest contrasts activated two dissociable networks. Most noticeably, whereas the recalling characteristics–rest contrast activated a bilateral network centered largely in the parietal and temporal lobes, the uses–rest contrast activated a network including the left IFG. We then examined the uses–recalling characteristics contrast following a night of normal sleep. This contrast revealed activations in the right middle temporal gyrus, cingulate gyrus, and left precuneus (**Table 1**).

**Table 1:** Coordinates for the activations reported in the text.

Analysis	Structure	*x*	*y*	*z*	*z-Score*	*k*
Uses–rest (normal sleep)	Medial frontal gyrus	-10	6	64	4.18	144
	Inferior frontal gyrus	-44	32	12	3.71	139
	Caudate	6	8	-12	3.68	59
	Lingual gyrus	14	-90	-8	3.63	44
Recalling characteristics–rest (normal sleep)		-20	-64	-10	5.09	254
	Parahippocampus	28	-54	-6	4.54	509
	Inferior parietal lobule	-56	-26	50	4.28	71
	Paracentral lobule	-10	-8	56	4.24	204
	Inferior parietal lobule	-58	-20	38	4.23	134
	Hippocampus	-28	-14	-22	4.08	392
	Precentral gyrus	-28	-16	64	4.01	68
	Fusiform gyrus	-28	-62	-20	3.92	212
	Middle temporal gyrus	-58	-22	-8	3.92	99
	Insula	36	-2	12	3.90	340
	Insula	-46	-12	16	3.80	61
	Superior temporal gyrus	28	4	-16	3.50	75
Uses–recalling characteristics (normal sleep)	Middle temporal gyrus	54	-10	-22	3.79	66
	Cingulate gyrus	0	-18	34	3.55	41
	Precuneus	-8	-62	32	3.49	47
Uses–rest (sleep deprivation)	Middle frontal gyrus	60	10	40	4.82	243
	Inferior frontal gyrus	-50	14	14	4.47	2476
	Middle temporal gyrus	48	-38	2	4.17	111
	Middle temporal gyrus	-58	-44	-14	4.06	617
	Middle frontal gyrus	-30	12	58	3.99	66
	Postcentral gyrus	62	-16	34	3.92	145
	Medial frontal gyrus	-8	10	56	3.91	399
	Middle frontal gyrus	-24	-6	56	3.82	102
	Medial frontal gyrus	-24	52	8	3.74	64
	Putamen	-18	12	2	3.57	239
	Cerebellum	34	-62	-30	3.47	55
Recalling characteristics–rest (sleep deprivation)	Medial frontal gyrus	-14	56	22	5.17	1435
	Insula	-48	-8	-6	4.73	832
	Middle temporal gyrus	-60	-52	-2	4.05	105
	Superior temporal gyrus	-46	-36	16	3.96	215
	Middle temporal gyrus	62	-52	-6	3.85	184
	Superior temporal gyrus	52	-50	18	3.77	642
	Culmen	14	-38	-28	3.76	95
	Superior occipital gyrus	38	-84	34	3.74	45
	Middle frontal gyrus	32	42	20	3.74	173
	Middle frontal gyrus	30	50	8	3.55	225
	Inferior parietal lobule	54	-54	46	3.30	73
Uses–recalling characteristics (sleep deprivation)	Inferior frontal gyrus	-52	24	10	3.51	73
Uses–recalling characteristics (sleep deprivation)*	Inferior frontal gyrus	-52	24	10	3.51	70
Uses–rest (sleep deprivation)–uses–rest (normal sleep)	Superior temporal gyrus	-58	-58	20	5.00	56
	Inferior frontal gyrus	-46	40	2	3.41	50
Recalling characteristics–rest (sleep deprivation)–recalling characteristics–rest (normal sleep)	No suprathreshold activation
Uses–recalling characteristics (sleep deprivation)–uses–recalling characteristics (normal sleep)	No suprathreshold activation

*k – cluster size (number of contiguous voxels), The coordinates are reported in MNI space.*

**FIGURE 3:**
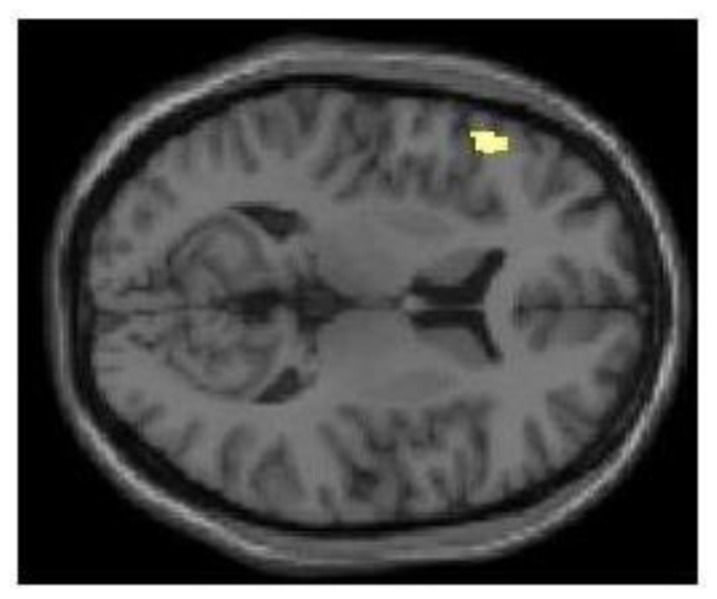
**Following sleep deprivation the left inferior frontal gyrus was activated more when generating uses (fluency) in the divergent thinking task.** Following sleep deprivation, there was greater activation in left inferior frontal gyrus when generating uses (compared to recalling characteristics) in the Alternate Uses Task. SPM rendered into standard stereotactic space and superimposed on to transverse MRI in standard space.

Next, we examined the uses–rest (ITI) and recalling characteristics–rest (ITI) contrasts, following sleep deprivation. Again, these contrasts demonstrated dissociable patterns of activation for generating uses vs. recalling characteristics (**Table 1**; **Figure 2**). Specifically, generating uses was associated with a primarily left-lateralized pattern of activation, with the largest cluster centered within the left IFG. In contrast, recalling characteristics was associated with a distributed bilateral pattern of activation involving the frontal, parietal, and temporal lobes. Then, we examined the uses–recalling characteristics contrast following sleep deprivation. This contrast revealed exclusive activation in the left IFG (**Table 1**; **Figure 3**).

To further isolate specific task-related activations underlying fluency and recalling characteristics under sleep deprivation vs. a night of normal sleep, we carried out three additional contrasts (of contrasts). The first involved uses–rest (sleep deprivation)–uses–rest (normal sleep), revealing activations in left superior temporal gyrus and IFG (**Table 1**). The second contrast involved recalling characteristics–rest (sleep deprivation)–recalling characteristics–rest (normal sleep), revealing no suprathreshold activation. Similarly, the third contrast involving uses–recalling characteristics (sleep deprivation)–uses–recalling characteristics (normal sleep) did not reveal any suprathreshold activation.

Finally, we examined whether RAPM, openness to experience, or CAQ had a significant effect on the activation patterns (when entered as covariates into the analysis). In each case, the results remained largely identical.

## DISCUSSION

The results demonstrated that our sleep deprivation intervention was effective in producing greater levels of fatigue and sleepiness in our participants compared to a night of normal sleep. Specifically, RT on PVT and ratings on SSS were progressively higher between 8 p.m. and 6 a.m. on the night of sleep deprivation, as were fatigue ratings on all five dimensions of MFI immediately prior to entry into the fMRI scanner in the morning following sleep deprivation. Furthermore, modeled cognitive effectiveness also exhibited significant reduction following sleep deprivation, suggesting that the participants’ capacity for engagement in cognitive tasks was diminished compared to a night of normal sleep. These results are necessary manipulation checks for interpreting our measures of interest.

Following a night of normal sleep, the uses–rest contrast revealed activation in a small network of regions shown previously to be activated in divergent thinking tasks (Chávez-Eakle et al., 2007; Fink et al., 2009; Abraham et al., 2012a; Kröger et al., 2012). Notable among the activated regions is left IFG, which was also shown to be activated in Fink et al.’s (2009) uses–fixation contrast using a similar paradigm. In turn, the recalling characteristics–rest contrast activated a bilateral network centered largely in the parietal and temporal lobes. Many of the activated regions in the temporal lobes – specifically those located in the medial temporal lobe (e.g., hippocampus and parahippocampus) – have been historically implicated in long-term memory (e.g., Squire and Zola-Morgan, 1991). Furthermore, the fusiform gyrus has been shown to contribute to the representation of object concepts in the brain (Martin, 2007). As such, their activation here is consistent with the requirements of the task (i.e., recollection of object characteristics). The uses–recalling characteristics contrast revealed activations in the right middle temporal gyrus, cingulate cortex, and left precuneus. Gonen-Yaacovi et al.’s (2013) recent large-scale meta-analysis demonstrated the reliable contributions of the middle temporal gyrus, the precuneus, and the cingulate gyrus across creativity tasks (see also Kleibeuker et al., 2013).

However, our focal interest in the present study consisted of examining patterns of activation in relation to fluency following sleep deprivation. The results demonstrated that following sleep deprivation, generating uses (compared to rest) was associated with a primarily left-lateralized pattern of activation, with the largest cluster centered within the left IFG. Much like the picture that emerged following a night of normal sleep, the pattern of brain activation in relation to generating uses was clearly dissociable from the pattern of brain activation in relation to recalling characteristics. Critically for testing our focal hypothesis, the uses–recalling characteristics contrast following sleep deprivation revealed exclusive activation in the left IFG. Our results suggest that the greater recruitment of PFC following short-term sleep deprivation – consistently observed in studies of verbal learning and logical reasoning – can be extended to fluency in divergent thinking. In other words, the elevated BOLD response in the left IFG might signal compensation due to sleep deprivation (Drummond et al., 2000, 2004, 2005; Jonelis et al., 2012).

The notable commonality across verbal learning, logical reasoning and divergent thinking tasks is that they all draw on WM and executive function, well known to engage lateral and inferior PFC. Perhaps not surprisingly, several recent studies have shown that divergent thinking ability (and creativity more broadly) draws heavily on executive function and WM (Sligh et al., 2005; Gilhooly et al., 2007; Nusbaum and Silvia, 2011; Beaty and Silvia, 2012; Benedek et al., 2012; De Dreu et al., 2012). Greater executive function and WM capacity have been shown to aid creative production in at least two ways. First, mechanistically, they increase one’s capacity to maintain and manipulate information in the focus of attention in the service of product generation. Second, motivationally, executive function and WM capacity enhance persistence, thereby minimizing undesirable mind wandering that would otherwise lead to premature cessation of problem solving (De Dreu et al., 2012). Evidence showing that impairment in fluency following sleep deprivation is accompanied by greater activation in left IFG is consistent with the account that executive function and WM likely play a role in response generation.

Our interpretation is also consistent with evidence regarding the role of IFG in controlled selection and retrieval of semantic information (e.g., Fink et al., 2009; Abraham et al., 2012a; Kröger et al., 2012). Strong evidence for this link was provided by Gonen-Yaacovi et al.’s (2013) meta-analysis which revealed that activation in a set of areas consisting of the left inferior frontal junction (BA 44/46) extending to dorsolateral PFC, left IFG (BA 45/47), and left angular gyrus (BA 39) was associated with generation *as well as* combination of remote ideas – both of which require the controlled selection and retrieval of semantic information. Those processes likely also draw on WM and executive function, such that the activation of IFG in the present study may reflect WM and executive function involvement in the service of selection and retrieval of semantic information.

Notably, the joint results of the three contrasts (of contrasts) conducted to isolate specific task-related activations support the conclusion that the activations observed in left IFG and superior temporal gyrus in relation to fluency under sleep deprivation are more likely due to general WM and executive function processes – including controlled selection and retrieval of semantic information – rather than more specific processes that distinguish fluency from recalling characteristics. Specifically, whereas the contrast of uses–rest (sleep deprivation)–uses–rest (normal sleep) revealed activations in left superior temporal gyrus and IFG, the contrast of uses–recalling characteristics (sleep deprivation)–uses–recalling characteristics (normal sleep) revealed no suprathreshold activation.

It is important to note that divergent thinking ability is not defined exclusively by executive function and WM capacity, despite the fact that they are necessary for establishing attentional control. In fact, evidence suggests that divergent thinking thrives as a function of flexible switching between focused and defocused modes of cognition as a function of task demands (Vartanian, 2009; Zabelina and Robinson, 2010; Wiley and Jarosz, 2012). The data presented here suggest that by disrupting sleep, one impairs fluency likely by disrupting the neural networks necessary for establishing attentional control. It will require additional experimentation to determine whether disrupting the neural networks that underlie defocused modes of cognition (e.g., the default network) will result in similar impairments in divergent thinking.

Interestingly, accounting for individual differences in fluid intelligence, openness to experience, and creative achievement did not change the magnitude of the response in PFC in fluency following short-term sleep deprivation. This result must be viewed with caution because our small sample size was not optimal for fully exploring the impact of individual differences on brain activation.

Related to this issue, the small sample size used in the present study represents a methodological limitation of our design. Although we used a liberal voxel-level criterion for reporting our results, all reported activations also survived a cluster-level correction of 40 contiguous voxels – four times the recommended minimum cluster size (i.e., 10) for selecting reliable activations (Lieberman and Cunningham, 2009; see also Forman et al., 1995). Because of our small sample size, the robustness of our findings must be determined in future replications.

Furthermore, we assessed performance on AUT only using *fluency*, represented by the total number of generated responses to a prompt. Although fluency accounts for a significant portion of the variance in divergent thinking tasks (Plucker and Renzulli, 1999), it is not itself a measure of creative cognition. In this sense, the results of the recent study by Kleibeuker et al. (2013) are particularly germane for interpreting our data. Their results demonstrated that the act of divergent thinking – when measured as a function of the production of multiple responses – recruits left PFC. In contrast, creative cognition in the context of a divergent thinking task recruits a more distributed network, including bilateral PFC. Because our participants were instructed to generate as many solutions as possible to prompts and we focused fully on fluency, the involvement of the left PFC exclusively in the contrast of interest (**Figure 3**) should be attributed to the demand to generate multiple responses rather than the demand to generate creative responses.

The results of the present study should be interpreted within the context of Lim and Dinges’ (2010) recent meta-analysis, which demonstrated that short-term sleep deprivation impairs a wide host of cognitive outcome variables including simple attention, complex attention, processing speed, WM, and short-term memory. Given that divergent thinking draws on many of these component processes – notably complex attention (i.e., executive function) and WM (Gilhooly et al., 2007; Beaty and Silvia, 2012; De Dreu et al., 2012) – its behavioral profile under short-term sleep deprivation demonstrates that divergent thinking performance will be affected by targeting its component processes. In addition, our results demonstrate that impairment in fluency following short-term sleep deprivation is likely to be instantiated in brain structures that underlie its component processes, in this case the left IFG.

## Conflict of Interest Statement

The authors declare that the research was conducted in the absence of any commercial or financial relationships that could be construed as a potential conflict of interest.

## Footnotes

1 Previous neuroscientific protocols involving data collection following nights of sleep deprivation and normal sleep have shown that when administered in counterbalanced order, a gap of 4–7 days is sufficient between sessions (e.g., Mograss et al., 2009).

2 Although dietary caffeine consumption *and* withdrawal are potential confounding variables in fMRI (Field et al., 2003), we opted to restrict consumption to minimize its potential mitigation of the effects of sleep deprivation on fatigue and sleepiness.

3 The dissociation between the generation phase and the response phase is implemented so that brain activation due to fluency is not confounded with brain activation due to the motor movement of pressing a button.

4 The characteristics block was included to mimic Fink et al.’s (2009) design as closely as possible. It is used as a control condition for recall from long-term memory. We did not test any hypotheses involving the effect of sleep deprivation on recall from long-term memory.

5 We were not able to collect complete SSS data from one subject.

6 Throughout the manuscript Cohen’s *d* is used as a measure of effect size for *t* tests. We used the following online calculator for calculating *d*: www.cognitiveflexibility.org/effectsize/

7 Self-reported data on the consumption of caffeine, nicotine, and alcohol were only available from nine participants. Cohen’s *d* for nicotine and alcohol could not be calculated because in both cases the average consumption following sleep deprivation was 0 (SD = 0).

8 The model underlying FAST^TM^ is SAFTE^TM^ (Sleep, Activity, Fatigue, and Task Effectiveness; Hursh et al., 2004). Specifically, FAST^TM^ is the interface for generating graphical representations of effectiveness scores, which are predictions derived from SAFTE^TM^.

9 Although unrelated to our *a priori* hypotheses, for interested readers we also calculated BFI scores for extraversion (*M* = 3.12, SD = 0.68), agreeableness (*M* = 3.91, SD = 0.69), conscientiousness (*M* = 3.91, SD = 0.37), and neuroticism (*M* = 2.42, SD = 0.59).
